# Comparative Analysis of Protein Structure Alignments

**DOI:** 10.1186/1472-6807-7-50

**Published:** 2007-07-26

**Authors:** Gabriele Mayr, Francisco S Domingues, Peter Lackner

**Affiliations:** 1 Department of Molecular Biology, University of Salzburg, Hellbrunnerstrasse 34, 5020 Salzburg, Austria; 2 Max-Planck-Institut für Informatik, Stuhlsatzenhausweg 85, 66123 Saarbrücken, Germany

## Abstract

**Background:**

Several methods are currently available for the comparison of protein structures. These methods have been analysed regarding the performance in the identification of structurally/evolutionary related proteins, but so far there has been less focus on the objective comparison between the alignments produced by different methods.

**Results:**

We analysed and compared the structural alignments obtained by different methods using three sets of pairs of structurally related proteins. The first set corresponds to 355 pairs of remote homologous proteins according to the SCOP database (ASTRAL40 set). The second set was derived from the SISYPHUS database and includes 69 protein pairs (SISY set). The third set consists of 40 pairs that are challenging to align (RIPC set). The alignment of pairs of this set requires indels of considerable number and size and some of the proteins are related by circular permutations, show extensive conformational variability or include repetitions. Two standard methods (CE and DALI) were applied to align the proteins in the ASTRAL40 set. The extent of structural similarity identified by both methods is highly correlated and the alignments from the two methods agree on average in more than half of the aligned positions. CE, DALI, as well as four additional methods (FATCAT, MATRAS, C_*α*_-match and SHEBA) were then compared using the SISY and RIPC sets. The accuracy of the alignments was assessed by comparison to reference alignments. The alignments generated by the different methods on average match more than half of the reference alignments in the SISY set. The alignments obtained in the more challenging RIPC set tend to differ considerably and match reference alignments less successfully than the SISY set alignments.

**Conclusion:**

The alignments produced by different methods tend to agree to a considerable extent, but the agreement is lower for the more challenging pairs. The results for the comparison to reference alignments are encouraging, but also indicate that there is still room for improvement.

## Background

Structural biology relies heavily on structure comparison methods. These methods are routinely applied in order to establish structural, evolutionary and functional relationships between proteins [1]. In general these methods provide a measure of structural similarity between proteins, which is used to identify similar folds and evolutionary related proteins. Most of the methods also generate an alignment that defines the residues that have a structurally equivalent role in the proteins compared. When the aligned proteins are assumed to share a common ancestor, a structure alignment supports the identification of evolutionary equivalent residues. Since protein structure is more conserved in evolution than sequence, structure alignments of remote homologous proteins are considered more reliable than sequence based alignments to identify the equivalent residues. The structure alignment of functionally related proteins provides insights into the functional mechanisms, and has been successfully applied in the functional annotation of proteins whose structures have been determined [2].

When aligning structures the nature of the structural models should also be taken into account. Experimental structural models are usually determined by X-ray crystallography or by Nuclear Magnetic Resonance spectroscopy. The atomic coordinates obtained from these experiments are always associated with some degree of uncertainty resulting from experimental errors and from the intrinsic flexibility of the proteins or from atom vibrations. These uncertainties become problematic especially for some comparison methods that assume that the protein backbone is formed by regular secondary structure elements, and correct assignment of these elements might not be possible for models with poor resolution. Additional difficulties originate from the nature of the protein structural relationships. Similar structures might display considerable structural variability and are often related by several insertions and deletions (indels) of considerable size. Structural variation is noticeable in the comparison of alternative conformations of a single protein, and reflects the intrinsic protein flexibility [3].

Structural similarity between different proteins is the result of evolution from a common ancestor if the proteins to be compared are homologous, or they are the result of convergent or parallel evolution [4]. The evolution of proteins involves mutations of single residues, insertions and deletions [5], gene duplication or fusion and exon duplication, deletion or shuffling [6]. Such changes accumulate over time and result in structural differences between the two proteins. These changes preferably affect the surface regions of the proteins, except for the functional sites which tend to be conserved if the protein retains the same molecular function. The hydrophobic core, essential to maintain structural integrity, in general remains relatively conserved [6,7]. Homologous proteins might also be related by circular permutation or shuffling of the protein sequence, which results in a non-sequential sequence or structure alignment between the two structures. Circular permutations are the result of gene duplication, exon shuffling or post-translation modifications [8].

Repetition is a common feature of protein structures, and is observed at different structural levels. These repetitions occur at the level of the secondary structure elements, at the level of supersecondary elements, at the subdomain level or at the domain level. Recurring substructures imply that protein structures can be aligned in alternative ways with comparable structural similarity scores. The existence of alternative alignments has been investigated before [9,10].

Currently there are a considerable number of structural comparison tools available to the structural biologist [1,11]). In general, these methods compare the geometry of the C_*α *_backbone atoms, but they are based on different algorithms and have been designed for various applications. CE [12] and DALI [13] are two popular methods for searching similarities in a structural database and for pairwise comparison of two structures. Both methods search for compatible pairs of fragments with similar intramolecular C_*α *_distances. Then they use different strategies to combine these fragments into a final alignment. Methods like FATCAT [14] are able to align subdomains in different relative orientations, resulting from protein flexibility or from evolutionary divergence [14]. Another strategy is to consider not only the backbone geometry but also the physicochemical environment of each residue in order to align the two structures [15-17]. This strategy is followed in the SHEBA implementation [15]. Some tools match secondary structure elements to obtain in an efficient way a first alignment that is later refined. MATRAS [17] in particular matches secondary structure elements in the first stage of alignment. Environmental properties and C_*α *_distances are then applied to obtain the final solution. MATRAS applies a Markov transition model of evolution to derive different types of scoring functions. Some methods, like C_*α*_-match, do not take the protein sequence order into account and allow for non-sequential alignments [18]. C_*α*_-match in particular is based on geometric hashing and ignores connectivity between aligned residues, which is desirable for the comparison of folds and architectures and for the comparison of proteins related by circular permutation. Other methods are able to align multiple structures [19-22]. Finally, some tools perform very fast comparisons between a given query protein and a structural database, and provide structural similarity scores for each comparison but no alignment [23,24].

So far structure comparison methods have been primarily evaluated in terms of their ability to identify proteins with similar folds or to identify homologous proteins [11,25,26]. They have also been assessed relative to the extent of structural similarity that is identified, where better performance corresponds to longer alignments and to better rigid body superpositions, or to a better score according to other geometric measures [26-29]. Several methods have been the focus of these analyses, in particular SSAP [30], STRUCTAL [31], DALI [13], LSQMAN [32], CE [12], SSM [29], ASH [27] and TM-align [28]. Less attention, however, has been given to the objective analysis of the extent of agreement between alignments produced by different pairwise structure comparison methods. There is also a need to assess the accuracy of these structure based alignments regarding the correct identification of equivalent residues in terms of structure, evolution or function.

We analysed and compared pairwise structure alignments produced by six methods based on different algorithms: CE, DALI, FATCAT, MATRAS, SHEBA and C_*α*_-match. First, CE and DALI were applied to a representative set of remote homologous proteins comprising 355 structure pairs derived from the ASTRAL database [33] (ASTRAL40 set). Then we applied CE, DALI, FATCAT, MATRAS, SHEBA and C_*α*_-match to 69 related protein pairs obtained from the SISYPHUS database [34] (SISY set). Finally, these six methods were applied to a third set comprising 40 pairs that are challenging to align. These pairs include **r**epetitions, **i**ndels, **p**ermutation and **c**onformational variability (RIPC set). The methods were compared in terms of the extent of structural similarity detected according to the resulting alignments and in terms of alignment consistency. The methods were also compared relative to the extent of agreement to reference alignments. Finally, to illustrate the different types of structure comparison challenges, the results of selected pairs were analysed in more detail.

## Results and Discussion

In this section we present the results for the comparison of CE and DALI structural alignments using the ASTRAL40 set. Then we provide the alignment comparison results obtained for the SISY and RIPC sets using six different methods. The alignments from different methods were compared regarding identification of structure similarity and alignment consistency (all sets) and agreement with reference alignments (for SISY and RIPC sets). Finally we describe in more detail the different alignments obtained for seven pairs of proteins from the RIPC set which illustrate the different types of challenges currently faced by structure alignment methods.

### ASTRAL40 structural alignments

The standard structure comparison methods CE and DALI were applied to each pair of structures in the ASTRAL40 set. Proteins of the ASTRAL40 collection are remote homologous in the sense that they have less than 40% sequence identity and belong to the same SCOP [35] superfamily but different families [see Additional file 1]. The structure based alignments obtained by CE and DALI were compared with regard to the identification of structural similarity and the consistency or agreement of the residues aligned.

#### Identification of structural similarity

Two standard measures of protein structure similarity can be derived from structure-based alignments: The alignment length expressed with the number of equivalent residues (EQR) and the root-mean-square distance (RMSD) of the superimposed structures. The number of equivalent residues provides a measure of how large is the region of structural similarity, and the RMSD provides a measure of the degree of structure similarity in the aligned region. The RMSD depends on the number of equivalent residues, therefore the RMSD values associated with alignments of different lengths can not be compared. Different normalised measures have been proposed [1]. In particular the RMSD_100 _corresponds to the RMSD value expected if the two protein structures were 100 residues length [36]. A simpler alternative is to divide the RMSD by EQR: RMSDN = RMSD/EQR.

**Figure 1:**
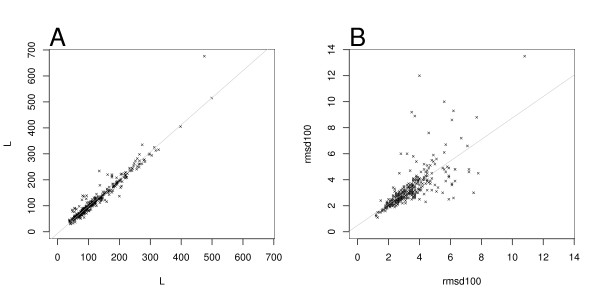
**Comparison of alignment length and RMSD_100 _for the ASTRAL40 set**. Alignments generated by CE are in the x-axis, DALI alignments in the y-axis and regression lines are in gray in both plots. A: Scatter plots for the alignment lengths. B: Scatter plots for RMSD_100 _values.

First we investigated the extent of the correlation between the alignments obtained with CE and with DALI relative to EQR and to RMSD_100_. Figure 1 shows the results for each pair of proteins in the ASTRAL40 set. The EQR are highly correlated, with a Pearson correlation coefficients of 0.97, and a Spearman correlation coefficient of 0.96. The RMSD_100 _values are less correlated, with 0.72 (Pearson) and 0.86 (Spearman).

In the ASTRAL40 set, RMSD_100 _values are highly correlated to the RMSDN (Pearson correlation 0.96). RMSD_100 _was selected for the analysis of the results. The differences in lengths and RMSD_100 _values for the alignments produced by CE and DALI for each pair are small in general. The CE alignments tend to be longer (median difference 3.0), while DALI alignments tend to have better RMSD_100 _values (median difference 0.1). Although the differences are small, the distributions of EQR and RMSD_100 _of the alignments obtained with CE and DALI are significantly different in the ASTRAL40 set according to the Wilcoxon signed-rank test with paired observations [37]. The p-values are 2.0·10^-8 ^for EQR and 3.0·10^-5^for RMSD_100_.

To summarise, the lengths of alignments produced by CE and DALI are highly correlated, but the RMSD_100 _values are less correlated. The differences between alignment lengths and the RMSD_100 _are small but significant, where DALI tends to generate shorter alignments but with better RMSD_100 _than CE.

#### Alignment consistency

**Figure 2:**
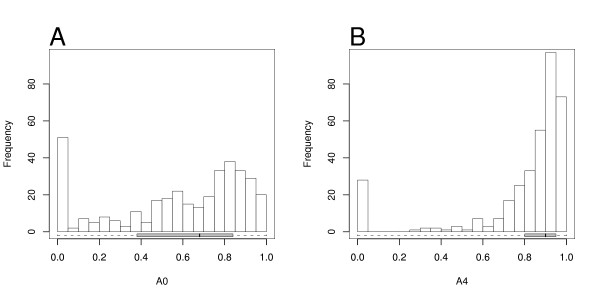
**Alignment consistency for the ASTRAL40 set**. Consistency between the alignments generated by CE and DALI for the ASTRAL40 set. Box-and-whisker plots are along the x-axis. A: Histogram of *A*_0 _values. B: Histogram of *A*_4 _values.

The extent of agreement between the residues aligned by CE and DALI was measured with *A*_0 _and *A*_4_, as described in the Methods section. The first measure is more strict as it only considers the matching aligned residues, while *A*_4 _tolerates shifts of up to four residues. Figure 2 provides the histograms for distributions of *A*_0 _and *A*_4 _values for the ASTRAL40 set.

The *A*_0 _distribution has mean of 0.59 and median 0.68. The more tolerant measure *A*_4 _has higher mean 0.80 and median 0.90, indicating that CE and DALI tend to align proteins in the same region. The spread is much larger for the *A*_0 _distribution. There is a maximum at [0.00, 0.05], with 14% of alignments having an *A*_0 _≤ 0.05, and there is a another peak around 0.8. The *A*_4 _shows a pronounced bimodal distribution with a peak at [0.90, 0.95], and a smaller peak at [0.00, 0.05]. For 8% of proteins CE and DALI alignments are completely different, with *A*_4 _≤ 0.05.

### Structural alignments from the SISY set

The SISY set is based on the SISYPHUS database, which contains structural alignments for proteins with non-trivial relationships [34]. Most pairs of the SISY set are categorised in SISYPHUS as homologous (52 out of 69) while the remaining are structurally related through a common fold or a fragment definition [see Additional file 2]. Alignments were calculated by CE, DALI, FATCAT, MATRAS, C_*α*_-match and SHEBA for each pair in the SISY set. These alignments were compared with regard to the extent of structural similarity detected and the consistency between alignments. The alignments obtained by the six different methods were also compared to reference alignments obtained from the SISYPHUS database.

#### Identification of structural similarity

We compared EQR of the alignments obtained with the six methods. There is a considerable correlation between all methods regarding the EQR [see Additional file 3, Figure S1]. In particular the correlation is high between CE and DALI, as observed previously with the ASTRAL40 set. MATRAS tends to show lower correlation with FATCAT and SHEBA. The correlation regarding RMSD_100 _is much lower. For example, between CE and DALI the correlation is 0.34 (Pearson) and 0.76 (Spearman).

The distribution of the differences of the length of the alignments generated by two methods for each pair of structures are given in Figure S2 in Additional file 3. In general SHEBA and FATCAT generate longer alignments than the other methods, while C_*α*_-match generates the shortest alignments. Similar analysis of RMSD_100 _differences indicates that C_*α*_-match has the smallest RMSD_100_, while SHEBA and to a less extent MATRAS alignments tend to have larger RMSD_100_.

#### Alignment consistency

**Figure 3:**
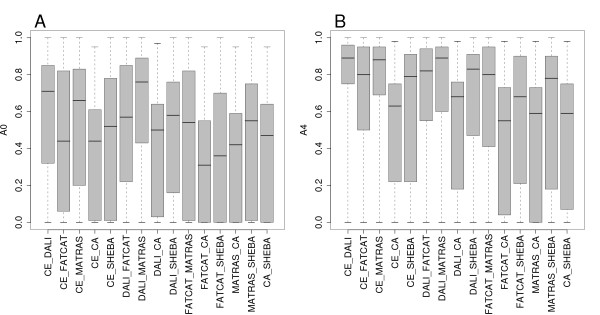
**Consistency of alignments between the six methods measured in the SISY set**. Box-and-whisker plots of the distributions of consistency values were computed for all 15 combinations of the six methods. CA stands for C_*α*_-match in all figure labels. A: *A*_0 _distribution. B: *A*_4 _distribution.

The consistency between alignments from different methods was measured according to *A*_0 _and *A*_4 _over the SISY set. The distributions of the *A*_0 _and *A*_4 _values for each pair of methods are shown in Figure 3 as box-and-whisker plots, the corresponding histograms are available in Figure S3 and Figure S4 in the Additional file 3. The spread of the *A*_0 _values is rather large. FATCAT and C_*α*_-match have the lowest alignment consistency according to *A*_0_. The best consistency according to *A*_0 _is observed between DALI and MATRAS, between CE and DALI, and between CE and MATRAS. The *A*_4 _distributions have considerably higher median values, but otherwise the trends are similar to the *A*_0 _results.

In order to group the methods according to the alignment consistency we used the mean values of *A*_0 _and *A*_4 _as well as the median values to compute several dissimilarity measures. Two dissimilarity measures were computed of the type *d *= 1 *- M*, where M is the mean of *A*_0 _or *A*_4_. Hierarchical clustering [38] was applied using the four alternative dissimilarity measures. The silhouette width value is a measure of cluster quality [39] and was applied to select the best number of clusters. The best average silhouette width values were obtained with two clusters using any of the two dissimilarity measures. One of the clusters included only the C_*α*_-match method and the remaining cluster included the other five methods. The silhouette width values were usually below 0.5. This indicates that the cluster quality is low, and that the methods are uniformly distinct regarding alignment consistency.

The alignment agreement between all six methods is in general much lower than between any pair of methods. When *A*_0 _is computed based on the number of aligned residues in common by all six methods, then the alignment consistency is 0.0 for 42%, and 64% have *A*_0 _≤ 0.20.

To summarise, the alignment agreement between two methods shows a large spread, and the observed range of medians is 0.3 *- *0.8. If alignment shifts of up to four residues are tolerated, then the range of medians increases to 0.5 *- *0.9. C_*α*_-match alignments tend to be less consistent with the alignments from other methods. The alignment agreement over all six methods is much lower, with 42% of the pairs sharing no aligned residues over all methods.

#### Comparison to reference alignments

**Figure 4:**
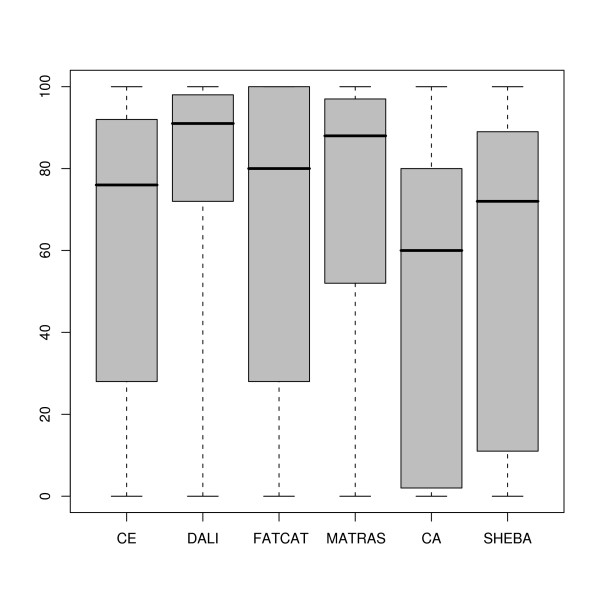
**Comparison to reference alignments in the SISY set**. Box-and-whisker plots of the distributions of the percentage of agreement to reference alignments obtained for each of the 6 methods.

The SISYPHUS database contains manually curated multiple structure alignments [34]. For each pair in the SISY set we extracted these reference alignments from the SISYPHUS database. To assess alignment accuracy, the alignments generated by the six methods were compared to these reference alignments, and the percentage of agreement was computed [see Additional file 4]. The corresponding box-and-wisker plots are available in Figure 4. The corresponding histograms are shown in Figure S5 in Additional file 3. The spread of accuracy values as measured by the agreement to SISYPHUS alignments is large for all methods except DALI. The lowest median accuracy value is 60% for C_*α*_-match (mean 51%), the largest median is obtained by DALI with 91% (mean 76%). Most methods have a peak of accuracy above 80% as shown in the histograms in Figures S5. The C_*α*_-match and SHEBA histograms show peaks at low accuracy values (below 20%). In four cases no method is able to align any residue correctly. For the pairs 1cr5C-1eu1A and 1okgA-1h9cA SISYPHUS defines short reference alignments (fragment category). The methods however generate larger global alignments which do not match these fragments aligned in SISYPHUS. In case of 1v7oA-1hr6B we observe circular permutations, and additionally the SISYPHUS target alignment does not superimpose very well. Finally, the structure 1gzdA contains two repeats of 1m3sA with several extensive indels.

**Figure 5:**
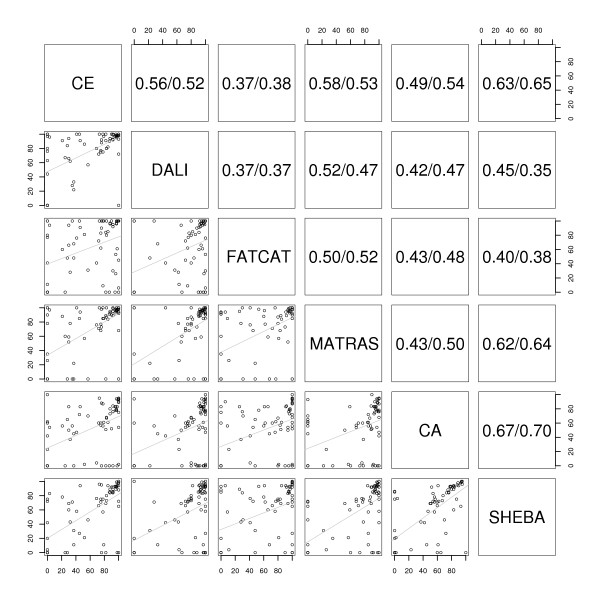
**Correlation for the comparison to reference alignments in the SISY set**. Analysis of the correlation between the alignments from different methods regarding the percentage of agreement to reference alignments. Lower left diagonal shows the scatter plots. Upper right diagonal shows the Pearson (first value) and Spearman (second value) correlation coefficients.

The distribution of the accuracy values from the six methods were compared using a two-sided Wilcoxon signed-rank test with paired observations [37]. The corresponding p-values are given in Table 1. DALI and CE alignment accuracy distributions are significantly different (p-value 3.8·10^-5^), with DALI accuracies having higher median and mean values. The results therefore indicate that DALI alignments agree better with SISYPHUS alignments than CE. In addition, the results in Table 1 show a significantly better agreement with SISYPHUS alignments for both DALI and MATRAS in comparison to SHEBA. The p-values in Table 1 also indicate that the agreement between C_*α*_-match alignments and SISYPHUS is significantly worse in comparison to DALI and MATRAS. Other differences are noticeable but they are not as significant (p-values around 10^-2^). The correlation between accuracy values was also investigated, and is in general very low (see Figure 5).

**Table 1:** Wilcoxon test for alignment accuracy in SISY set.

	DALI	FATCAT	MATRAS	C_*α*_-match	SHEBA
CE	3.8·10^-5^	2.7·10^-1^	1.5·10^-2^	6.7·10^-2^	2.6·10^-1^
DALI		1.4·10^-2^	1.2·10^-2^	2.2·10^-8^	9.6·10^-7^
FATCAT			3.6·10^-1^	9.4·10^-3^	1.4·10^-1^
MATRAS				5.6·10^-5^	7.0·10^-4^
C_*α*_-match					9.2·10^-3^

### Structural alignments from the RIPC set

In order to improve current methods, a better understanding of their limitations is required. This can be provided by the analysis of structurally related proteins that are problematic to align, which are available in the RIPC set. The RIPC set comprises 40 structure pairs (Table 2 and Additional file 5). The pairs in this set are difficult to align as they include repetitions, extensive indels, circular permutations and/or considerable conformational variability. We compared the alignments calculated by CE, DALI, FATCAT, MATRAS, C_*α*_-match and SHEBA. Reference alignments were derived for a subset of the RIPC set based on sequence and function conservation. These reference alignments were used to investigate the ability of methods to correctly align functional residues and alternative conformers of flexible proteins.

**Table 2:** List of pairs in RIPC set

	SCOP Domains		Lengths			SCOP Classification		
Type	Dom1	Dom2	L1	L2	SeqID	Dom1	Dom2	Ref
I R	d1he9a_	d1nfn__	134	132	22.6	a.24.11.1	a.24.1.1	no
I C	d1hcy_2	d1lnlb1	263	307	13.9	a.86.1.1	a.86.1.1	yes
I R	d1afra_	d1jkua_	345	266	19.2	a.25.1.2	a.25.1.3	no
I	d1ay9b_	d1b12a_	108	239	20.0	b.87.1.1	b.87.1.2	yes
I	d1hx6a1	d1p2za2	230	312	16.5	b.121.2.1	b.121.2.2	no
I	d1b09a_	d1dy4a_	206	434	18.4	b.29.1.5	b.29.1.10	no
I R	d1olza2	d2trcb_	474	340	17.8	b.69.12.1	b.69.4.1	no
I	d1gbg__	d1ovwa_	214	398	19.5	b.29.1.2	b.29.1.10	yes
I	d1ed9a_	d1p49a_	449	548	18.8	c.76.1.1	c.76.1.2	no
I R	d1xyza_	d2hvm__	320	273	17.7	c.1.8.3	c.1.8.5	no
I	d1crl__	d1ede__	534	310	22.0	c.69.1.17	c.69.1.8	yes
I R	d1cpo_1	d1cpo_2	120	179	14.8	a.39.3.1	a.39.3.1	no
I C	d1jj7a_	d1lvga_	251	190	21.1	c.37.1.12	c.37.1.1	yes
I	d2adma_	d2hmyb_	386	327	15.3	c.66.1.27	c.66.1.26	yes
I C	d1lzy__	d148le_	129	162	18.1	d.2.1.2	d.2.1.3	no
I	d1an9a1	d1npx_1	247	198	19.3	c.4.1.2	c.3.1.5	yes
I C	d1dmaa_	d1lt3a_	204	226	21.3	d.166.1.1	d.166.1.1	no
I	d1bmld3	d2sak__	88	121	22.2	d.15.5.1	d.15.5.1	no
I	d1ng4a2	d3cox_2	88	130	17.7	d.16.1.3	d.16.1.1	no
I	d1aqza_	d1a2pa_	142	108	16.0	d.1.1.3	d.1.1.2	no
P R	d1nk1__	d1qdma1	78	77	24.3	a.64.1.1	a.64.1.2	yes
P	d1d5ra1	d1rsy__	133	135	21.8	b.7.1.1	b.7.1.2	no
P	d1nls__	d2bqpa_	237	228	43.9	b.29.1.1	b.29.1.1	yes
P	d1qasa2	d1rsy__	126	135	26.4	b.7.1.1	b.7.1.2	yes
P C	d1b6a_1	d1bia_1	74	63	25.7	a.4.5.25	a.4.5.1	no
P R	d1b5ta_	d1k87a2	275	351	17.5	c.1.23.1	c.1.23.2	yes
P I	d1jwyb_	d1puja_	281	261	20.2	c.37.1.8	c.37.1.8	yes
P I	d1jwyb_	d1u0la2	281	212	19.5	c.37.1.8	c.37.1.8	yes
P I	d1nw5a_	d2adma_	270	386	16.4	c.66.1.11	c.66.1.27	yes
P C	d1gsa_1	d2hgsa1	122	102	15.6	c.30.1.3	c.30.1.4	yes
P I	d1qq5a_	d3chy__	245	128	20.2	c.108.1.1	c.23.1.1	yes
P I	d1kiaa_	d1nw5a_	275	270	19.9	c.66.1.5	c.66.1.11	yes
C R	d1aj3__	d2spca_	98	107	22.2	a.7.1.1	a.7.1.1	no
C R	d2bbma_	d4cln__	148	148	100.0	a.39.1.5	a.39.1.5	yes
C R	d1dlia1	d1mv8a1	98	98	26.3	a.100.1.4	a.100.1.4	yes
I C	d1hava_	d1kxf__	216	159	20.1	b.47.1.4	b.47.1.3	yes
C	d1l5ba_	d1l5ea_	101	101	100.0	b.89.1.1	b.89.1.1	yes
I C	d1adl__	d1mup__	131	157	14.1	b.60.1.2	b.60.1.1	no
C	d1ggga_	d1wdna_	220	223	100.0	c.94.1.1	c.94.1.1	yes
I C	d1d5fa_	d1nd7a_	350	374	34.6	d.148.1.1	d.148.1.1	yes

Type: Type of difficulty for alignment algorithms. R: Repetitions, I: Insertions, P: Permutation, C: Conformational changes. SeqID: Sequence identity in percent. Ref: A reference alignment is available to evaluate alignment accuracy.

#### Structural similarity and alignment consistency

The results for the comparison of the alignment EQR and RMSD100 obtained in the RIPC set are similar to the results obtained in the SISY set. In particular there is considerable correlation of EQR values between alignments from different methods. The correlation for RMSD100 is also much lower.

**Figure 6:**
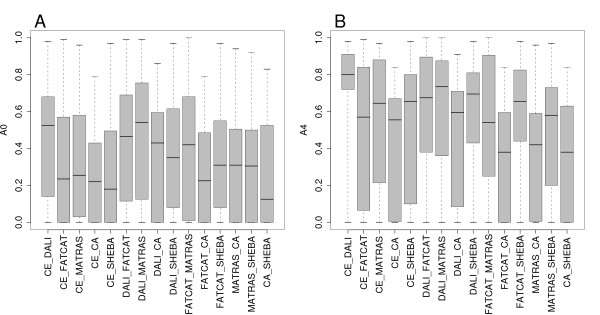
**Consistency of alignments between the six methods measured in the RIPC set**. Box-and-whisker plots of the distributions of consistency values were computed for all 15 combinations of the six methods. A: *A*_0 _distribution. B: *A*_4 _distribution.

The consistency between alignments from different methods was measured according to *A*_0 _and *A*_4 _over the RIPC set. The distributions of the *A*_0 _and *A*_4 _values for each pair of methods are shown in Figure 6 as box-and-whisker plots. The alignment consistency tends to be lower than the one observed in the SISY set, but otherwise the trends are similar. C_*α*_-match and SHEBA have the lowest alignment consistency according to *A*_0_. The best consistency according to *A*_0 _is observed between CE and DALI, and between DALI and MATRAS. In general C_*α*_-match alignments tend to have low *A*_0 _values when compared to alignments from other methods.

#### Comparison to reference alignments

**Figure 7:**
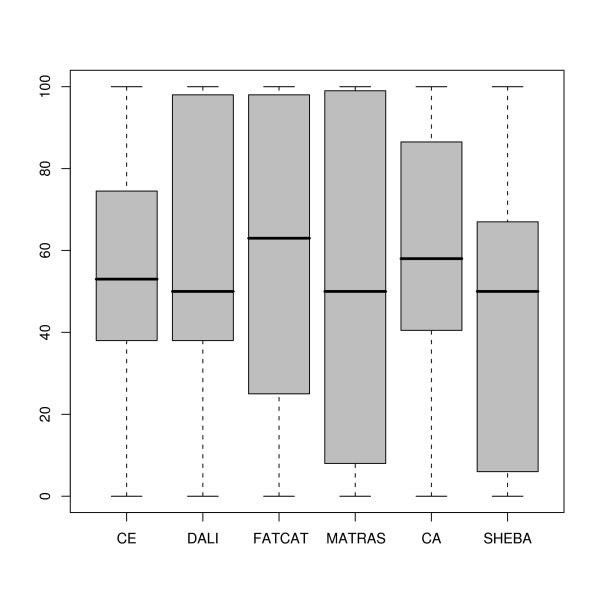
**Comparison to reference alignments in the RIPC set**. Box-and-whisker plots of the distributions of the percentage of agreement to reference alignments obtained for each of the 6 methods.

We derived reference alignments for 23 pairs of the RIPC set that correspond to pairs of residues that are expected to be equivalent as described in the Methods section. As with the SISY set, the alignment accuracy was measured by comparison of the reference alignments to the alignments obtained with the six methods [see Additional file 6]. The results obtained for each method are compared in Figure 7. The spread of accuracy values is large for all methods. The mean and median accuracy values are around 50% for most methods and lower than observed in the SISY set. The distribution of the accuracy values from the six methods were compared using a Wilcoxon paired test. In general, no significant difference between the distributions according is observed (p-values above 0.1). The only two exceptions, although the statistical significance is still low (with p-values 0.050), are the comparison between FATCAT and SHEBA and between DALI and SHEBA.

### Alternative alignments

So far we have investigated the consistency between alignments from different methods. There are two possible reasons for the differences between alignments. First, only one of the two alignments is optimal in the sense that it identifies the regions with most extensive structural similarity, or it identifies the evolutionary equivalent residues. Second, different alignments are equally optimal which is possible if there is not one but several alternative solutions for aligning two proteins. Such different alignments result usually from the existence of repetitions in the structures compared. They correspond to the same degree of structural similarity or to alternative ways to define evolutionary equivalent residues.

Some of the structure comparison methods produce alternative solutions, but in the previous analysis we only considered one single alignment from each method (the best scoring alignment). This might result in low consistency between alignments for pairs of structures that have alternative alignments. To investigate the role of alternative optimal alignments, one can consider all the alternative alignments from the different methods. Another simpler approach is to remove from the sets the pairs for which a method gives alternative alignments, and then investigate whether the consistency improves for the remaining pairs with unique alignments. We decided for the second approach, and removed from the ASTRAL40, SISY and RIPC sets all pairs with alternative solutions according to DALI. In total 124 pairs were excluded from the ASTRAL40 set (231 pairs remaining), 21 pairs were excluded from the SISY set (48 remaining), and 14 pairs were removed from the RIPC set (26 remaining). The consistency scores *A*_0 _and *A*_4 _were recomputed for these subsets. The new results show an improvement (better agreement) in some cases, but in general they are still similar to the ones obtained with the original sets. This indicates that the methods actually produce different solutions for some pairs, independent of the existence of alternative alignments.

### Analysis of selected challenging examples

In this section we investigate seven pairs of SCOP domains selected from the RIPC set. These examples illustrate how repetitions, indels, circular permutations and local conformational changes affect structural alignment results. For each example alignment path plots are provided for the visualisation of the alignments from the different methods.

#### Repetitions

**Figure 8:**
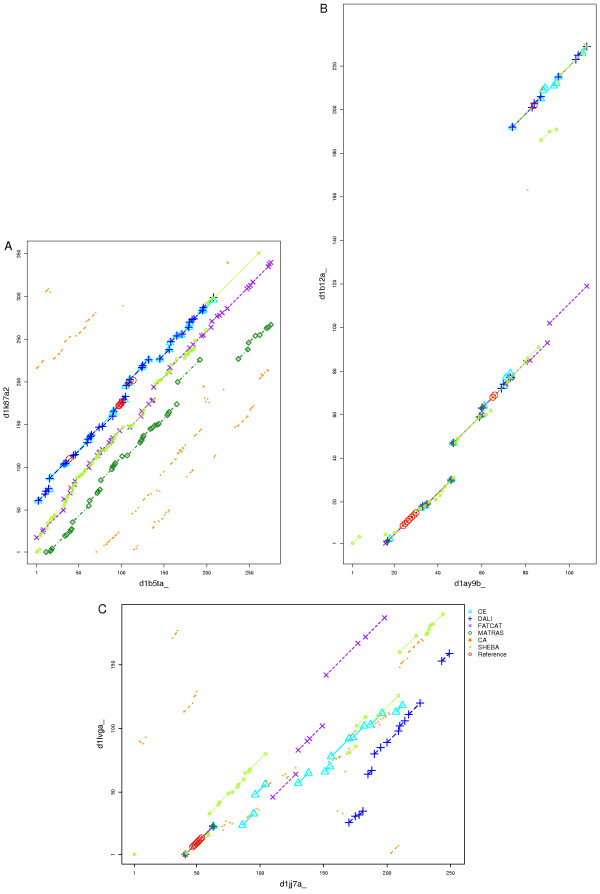
**Alignment paths from 6 methods including reference positions**. The x-axis corresponds to the residue positions in the backbone of one of the structures and the y-axis corresponds to the residue positions in the second structure. Aligned fragments are shown as lines with symbols at start and end of the aligned fragment pairs. Single aligned residues are plotted as symbols. The alignment of each method is represented by a specified symbol, line style and colour code. Reference positions are shown as red circles. A: Alignment plot of TIM barrel domains d1b5ta and d1k87a2. The different alignments result from the repetitive motifs in the TIM barrel fold and from the circular permutation. B: Signal peptidases d1ay9b_ and d1b12a_. There is a large insertion in the Type I signal peptidase d1b12a_. C: Alignment plot of P-loop containing NTP hydrolases d1jj7a_ [63] and d1lvga_ [64]. There are many indels of different sizes in the alignments.

Figure 8A shows the alignments of two FAD-linked oxidoreductases, Methylenetetrahydrofolate reductase (d1b5ta_) [40] and the proline dehydrogenase domain of bifunctional PutA protein (d1k87a2) [41]. The proteins have a TIM barrel fold which consists of repetitive *α*/*β *supersecondary motifs arranged in a closed barrel. Given the repetitive supersecondary motifs many alternative alignments are possible. In addition, the two proteins are related by circular permutation. Given the combination of repetitive elements and circular permutation it is not surprising that the differences between the alignments from the six methods are considerable. The methods match differently the repetitive *α*/*β *motifs. The result is parallel alignment paths that are shown in Figure 8A. They are separated in general by ~30 residues, the distance between the consecutive *α*/*β *motifs. DALI and CE are the only methods that correctly align the N-terminal region until the circular permutation point, approximately at position 220 in d1b5ta_. The two alignments overlap extensively (more than 163 equivalent residues) and succeed in matching five out of the eight amino acids in the reference alignment that are involved in binding FAD. No method correctly aligns the C-terminal region of d1b5ta_ to the N-terminal region of d1k87a2.

#### Indels

**Figure 9:**
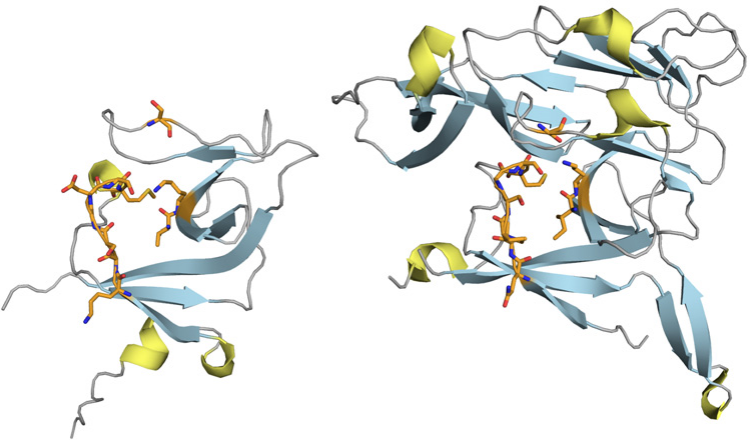
**Structures of Umud' and Type I signal peptidase**. E.coli Umud' (SCOP d1ay9a_) [65] is on the left and E.coli Type I signal peptidase (SCOP d1b12a_ [42]) is on the right. The amino acids included in the reference alignment are represented as orange sticks, helices in yellow, strands in blue.

The Umud protein is activated to Umud' by a RecA activated self-cleavage process that removes the N-terminal fragment with 24 residues. Umud' is involved in the SOS DNA-repair response in *E. coli*. The resemblance to a domain of SPase, a type I signal peptidase, has been observed [42]. This shared domain includes the catalytic residues essential for protease function that are conserved in both proteins. The SPase contains an additional all-*β *subdomain inserted in the conserved domain [42]. The structures are visualised in Figure 9. Figure 8B shows the corresponding alignment paths. The ten reference alignment residues are clustered in three different regions along the backbone, and include two catalytic site residues. Most of Umud' can be aligned, but a large gap is required to accommodate the subdomain inserted in the SPase and correctly align the C-terminal region. This correct alignment is achieved by most methods, which also succeed in matching the reference alignment. Some methods failed to place the required large gap and therefore incorrectly aligned the C-terminal region. In particular FATCAT introduces two twists and incorrectly aligns parts of the inserted subdomain.

P-loop containing NTP hydrolases are difficult cases for alignment because they may vary in the number of *β*-strands in the central sheet. Aligning these proteins therefore requires several indels [6]. Figure 8C shows extensive differences in the alignments by the different methods of the two NTP hydrolases. The N-terminal P-loop, the region associated with ADP binding, is in general correctly aligned by the different methods.

#### Circular permutations

**Figure 10:**
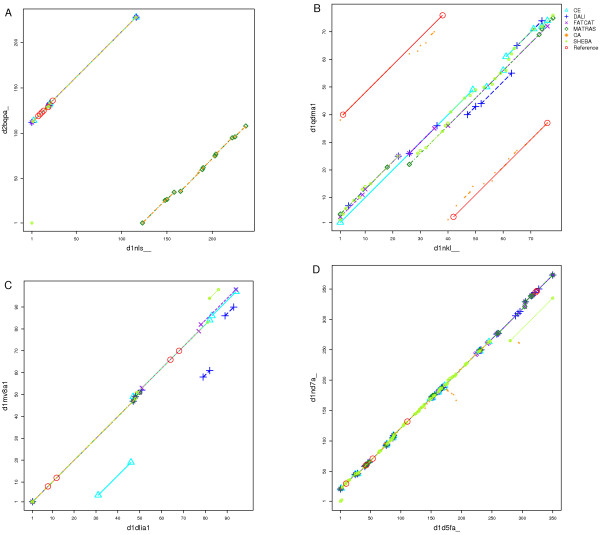
**Alignment paths from 6 methods including reference positions**. Alignments are represented as in Figure 8. A: Concanavalin A d1nls__ aligned with legume lectin d2bqpa_. All the applied methods except C_*α*_-match align either N or C termini of the domains. The consistency between the methods is high. B: Alignments of saposins NK-lysin d1nkl__ with swaposin d1qdma1. All methods align the structures in sequence order except C_*α*_-match. C: Alignments of UDPGDH middle domain d1dlia1 and GDP-mannose 6-dehydrogenase middle domain d1mv8a1. These two helical structures show considerate conformational differences. D: Alignment plot of Hect domains d1d5fa_ and d1nd7a_. The proteins consist of two subdomains in different relative orientations.

A prominent example for circular permutation is Concanavalin A. This protein has a *β*-sandwich structure (d1nls__ [43]), and is posttranslationally cleaved resulting in new N- and C-termini. Pea lectin (d2bqpa _[44]) resembles d1nls__ but is not processed in the same way, therefore the N-terminal region in one protein matches the C-terminal region in the other and vice versa. Figure 10A shows two aligned fragments, corresponding to the alignment of the two N to C-terminal regions that is characteristic of circular permuted proteins. Most methods align only the N-terminus half of Concanavalin A to the C-terminus of pea lectin (upper right path). MATRAS matches the other region, aligning the C-terminal of Concanavalin A to N-terminal of pea lectin (lower right). Only C_*α*_-match correctly aligns the complete structures. All methods (except MATRAS) share 104 aligned residues. The reference alignment includes six residues involved in *Ca*^2+ ^and *Mn*^2+ ^binding. Most methods correctly align five of these binding residues, and SHEBA succeeds in aligning all of them.

The homology between NK-lysin and swaposin with a circular permutation was revealed by sequence prior to knowledge of the crystal structures. The common fold consists of five helices forming a folded leaf. A previous curated alignment of the two proteins [6] was employed as reference alignment. Figure 10B shows that the methods align the helices in sequence order, which is incorrect regarding the evolutionary equivalent residues, and results in poor RMSD_100 _values (around 4). The exception is the alignment from C_*α*_-match, which aligns some residues in the permutated regions correctly, although most of the equivalences are sequentially unconnected. No aligned residues are shared by all six methods.

#### Conformational variability

The UDP-glucose-6-dehydrogenase middle domain d1dlia1 [45] and the GDP-mannose-6-dehydrogenase middle domain d1mv8a1 [46] are structurally related and of similar size. The Catalytic Site Atlas identifies four equivalent catalytic residues that are used to define the reference alignment. The two proteins consist of two common substructures that are conserved but in considerably different relative orientations. Most methods align only the N-terminal region, but CE matches only the C-terminal fragment. We observe a high alignment consistency in the first substructure located in the N-terminal region (see Figure 10C). FATCAT and SHEBA succeed in aligning the two substructures and the catalytic amino acids correctly.

**Figure 11:**
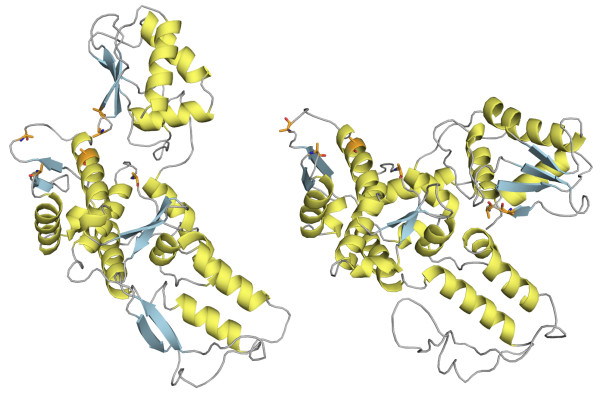
**Structures of Hect domains from E3 from ubiquitin-protein ligase E3a and WW domain-containing protein 1**. Hect domains from E3a (SCOP d1d5fa_) is on the left and protein 1 (SCOP d1nd7a_) is on the right. The amino acids included in the reference alignment are represented as orange sticks, helices in yellow, strands in blue.

The Hect E3 ligase catalytic domain consists of two *α*+*β *subdomains. This domain is present in the Ubiquitin-protein ligase E3a (d1d5fa _) [47] and in the WW domain-containing protein 1 (d1nd7a _) [48]. The domains in the two proteins share 35% sequence identity. The subdomains are conserved in the two proteins but in different relative orientations (see Figure 11). We defined a reference alignment based on six catalytic site residues. The alignment consistency between the different methods is shown in Figure 10D. The overlapping alignment paths indicate a high consistency between the alignments from different methods in the N-terminal region corresponding to the first subdomain. In total 97 positions are equally matched by all methods. FATCAT, MATRAS and DALI also correctly align the second subdomain that corresponds to 100 residues in the C-terminal region. All methods correctly align some of the functional residues, but only FATCAT succeeds in all six residues.

## Conclusion

We have presented a comparative analysis of pairwise structural alignments using three different datasets. The aim of this work is not to rank or benchmark the different methods, but instead to reveal the differences in the results and the challenges these methods face. The results indicate that the alignments of homologous proteins generated by two standard methods (DALI and CE) tend to be similar. Nevertheless for some of these homologous pairs the alignments are still completely different. The alignment agreement is lower in the more challenging datasets (SISY and RIPC sets), in particular if alignments from other methods (FATCAT, MATRAS, C_*α*_-match and SHEBA) are also compared. For these two datasets, reference alignments were compiled based on curated alignments and on the identification of equivalent functional residues. We find that the different methods tend to match the reference alignments to some extent, but there is still large room for improvement, specially for the more challenging protein pairs.

The analysis of the results obtained for seven protein pairs that are challenging to align illustrated the strengths and limitations of the different methods. These examples revealed how repetition and extensive indels results in low alignment consistency. They also revealed how proteins related by circular permutations are still difficult to align correctly by most methods, and that some methods can successfully align proteins with considerable conformational variability.

These results raise several issues of relevance for the users of structure alignment methods. In particular the results indicate that different alignments can be obtained when comparing the structures of remote homologous proteins with different methods. In addition, the resulting alignments not always match equivalent functional residues or curated alignments. These findings should also encourage developers to further improve their methods. In particular they should focus on testing and improving the results for challenging cases, as provided in the RIPC set.

The current study focused on the analysis of pairwise structure alignments. It would be of interest to perform in the future a similar comparative analysis of multiple structure alignments. In this respect one should take into account the procedures that have been successfully established to test multiple sequence alignment tools [49-51].

## Methods

### Datasets

#### Collection of the ASTRAL40 dataset

SCOP domains with less than 40% sequence identity were derived from the ASTRAL compendium. From every superfamily, a representative from each of two different randomly chosen families were randomly selected. Multichain domains were excluded. The ASTRAL40 set contains 355 structure pairs, and is available in the Additional file 1.

#### Collection of the SISY dataset

From each SISYPHUS multiple structure alignment [34] the pair of proteins with the lowest identity was chosen. Pairs with more than 40% sequence identity or including structures comprised of multiple chains were excluded. The full protein chains were used to generate the alignments. Reference alignments were obtained from the SISYPHUS database as well. The SISY set comprises 69 structure pairs. 52 pairs are grouped in the SISYPHUS homologous category. The dataset is available in the Additional file 2.

#### Assembly of the RIPC dataset

The RIPC set was collected by consulting the SCOP classification of proteins for remote homologous structure pairs and the Molecular Movements Database [52] for proteins with alternate conformations. We paid attention that all-*α*, all-*β *and *α*/*β*-containing domains are represented. The resulting set comprises 40 protein pairs. Each pair is associated with at least one of the structure comparison challenges: Repetitions, Indels, circular Permutation and extensive Conformational differences. The dataset is available in Table 1 and in the Additional file 5.

#### Calculation of sequence identity

Sequence identity was calculated using JAligner, an open source Java implementation of the Smith-Waterman algorithm [53,54] with following parameters: PAM250, open penalty 8 and extension 1.

### Comparison of structural alignments

#### Application of structure alignment methods

We applied CE and DALI to align the protein pairs in the ASTRAL40 set. CE, DALI, FATCAT, MATRAS, C_*α*_-match and SHEBA were applied to align the protein pairs in the SISY and RIPC sets. The standalone implementations of CE, DALI and SHEBA were used, FATCAT, MATRAS and C_*α*_-match results were obtained by accessing the corresponding online services [55-57]. Python scripts where implemented to parse the output files.

#### Alignment consistency

The alignment consistency score *A*_*s *_expresses the relative similarity of two alignments:

As=IsLmax
 MathType@MTEF@5@5@+=feaafiart1ev1aaatCvAUfKttLearuWrP9MDH5MBPbIqV92AaeXatLxBI9gBaebbnrfifHhDYfgasaacH8akY=wiFfYdH8Gipec8Eeeu0xXdbba9frFj0=OqFfea0dXdd9vqai=hGuQ8kuc9pgc9s8qqaq=dirpe0xb9q8qiLsFr0=vr0=vr0dc8meaabaqaciaacaGaaeqabaqabeGadaaakeaacqWGbbqqdaWgaaWcbaGaem4Camhabeaakiabg2da9maalaaabaGaemysaK0aaSbaaSqaaiabdohaZbqabaaakeaacqWGmbatdaWgaaWcbaGaemyBa0MaemyyaeMaemiEaGhabeaaaaaaaa@38A6@

where *I*_*s *_is the number of aligned residues that are consistent in the two alignments within a tolerance shift of *s *positions. Therefore *I*_0 _is the number of identically aligned residues in the two alignments, *I*_1 _is *I*_0 _plus the number of aligned residues that are shifted by one position, *I*_2 _is the number of aligned residues within a shift of two, and so on. *L*_*max *_is the length of the longer structure alignment: *L*_*max *_= *max*(*L*_1_*, L*_2_).

In the current work *A*_0 _is used to measure the extent of identity between alignments, and *A*_4 _is used to measure the extent of similarity between alignments. A value of *s *= 4 tolerates shifts of four aligned positions, corresponding to consecutive turns in an *α*-helix. *A*_*s *_values range between 0, corresponding to no similarity, and 1, where all aligned residues in the two alignments are consistent within shift *s*.

#### Comparison to reference alignments

The agreement to reference alignments was computed as the percentage of residues aligned identically to the reference alignment (*I*_*s*_) relative to the length of the reference alignment (*L*_*ref*_): 100·*I*_*s*_/*L*_*ref*_

### Definition of reference alignments in RIPC

Reference alignments were derived for 23 pairs from the RIPC set. Two of these are based on curated alignments of homologous proteins [6]. Three additional reference alignments result from mapping the residue numbers in the PDB structures that correspond to alternative conformations of the same protein. The remaining 18 reference alignments are the result of a search for functionally equivalent residues. Reference alignments are available in the Additional file 7.

Several strategies were applied in the search for functionally equivalent residues. If the two proteins bind the same or similar ligand then SiteEngine [58] was applied to confirm that these binding sites share similar physicochemical environments and similar structures and to obtain the equivalent residues. Equivalent catalytic residues or metal binding sites were obtained from the Catalytic Site Atlas [59,60], from the PDBSum [61] or from the literature. SPASM [62] was then applied to verify that the geometry is conserved in these sites.

## Authors' contributions

GM derived the datasets, computed and interpreted the results and drafted the manuscript. FSD contributed to the design of the study and to the computation and interpretation of results. PL conceived and designed the study and contributed to the computation and interpretation of results. All authors contributed to the writing of the manuscript, and read and approved the final version.

## Supplementary Material

Additional file 1 The ASTRAL40 dataset. Click here for file

Additional file 2 The SISY dataset. Click here for file

Additional file 3 Figures S1, S2, S3, S4, S5. Click here for file

Additional file 4 Table of SISY set alignment accuracy. Alignment accuracy in percentage of number of aligned reference positions. Mol1, Mol2: PDB code and chain identifier of the aligned molecules. Ref: Length of reference alignment. Click here for file

Additional file 5 The RIPC dataset. Type: Structure alignment problem of a pair (R: repetition, I: indels, P: permutation, C: conformational variability). Click here for file

Additional file 6 Table of RIPC set alignment accuracy. Alignment accuracy in percentage of number of aligned reference positions. Type: Structure alignment problem of a pair (R:repetition, I: indels, P: permutation, C: conformational variability). Ref: Length of reference alignment. Click here for file

Additional file 7 Reference alignments for the RIPC set. Click here for file
