# Supplementary material for: Comparative Analysis of Protein Structure Alignments
Source: BMC Struct Biol. 2007 Jul 26;7:50. doi: 10.1186/1472-6807-7-50 (PMC1959231; doi:10.1186/1472-6807-7-50)
Supplement: Additional file 3 — Figures S1, S2, S3, S4, S5. [file 1472-6807-7-50-S3.pdf]

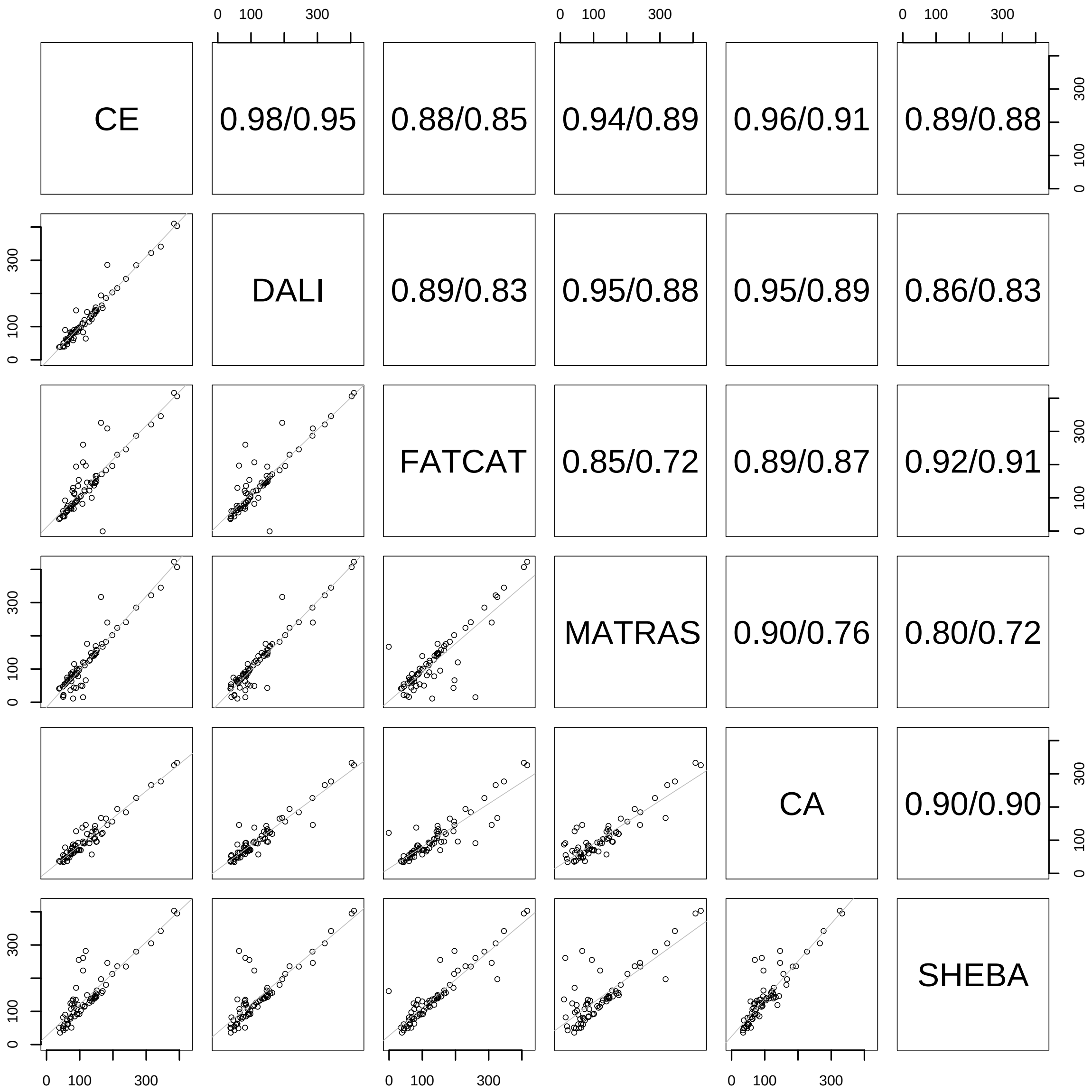

Figure S1: Comparison of lengths of alignments from the six methods for the SISY set. Lower left diagonal shows the scatter plots. Upper right diagonal shows the Pearson (first value) and Spearman (second value) correlation coefficients.

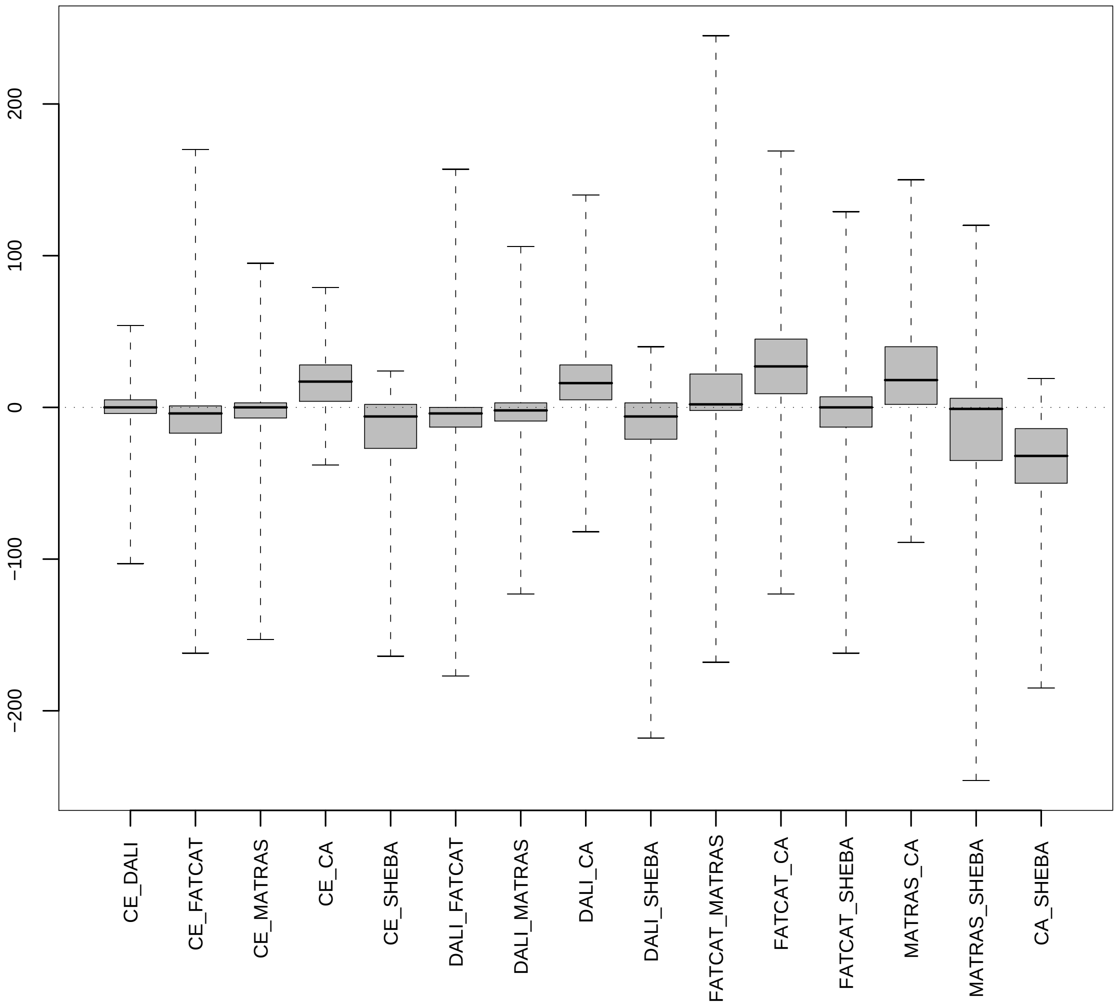

Figure S2: Box-and-whisker plots of the distribution of the differences in alignment lengths in the SISY set. For each pair in the SISY set the differences between the alignments from two methods are computed.

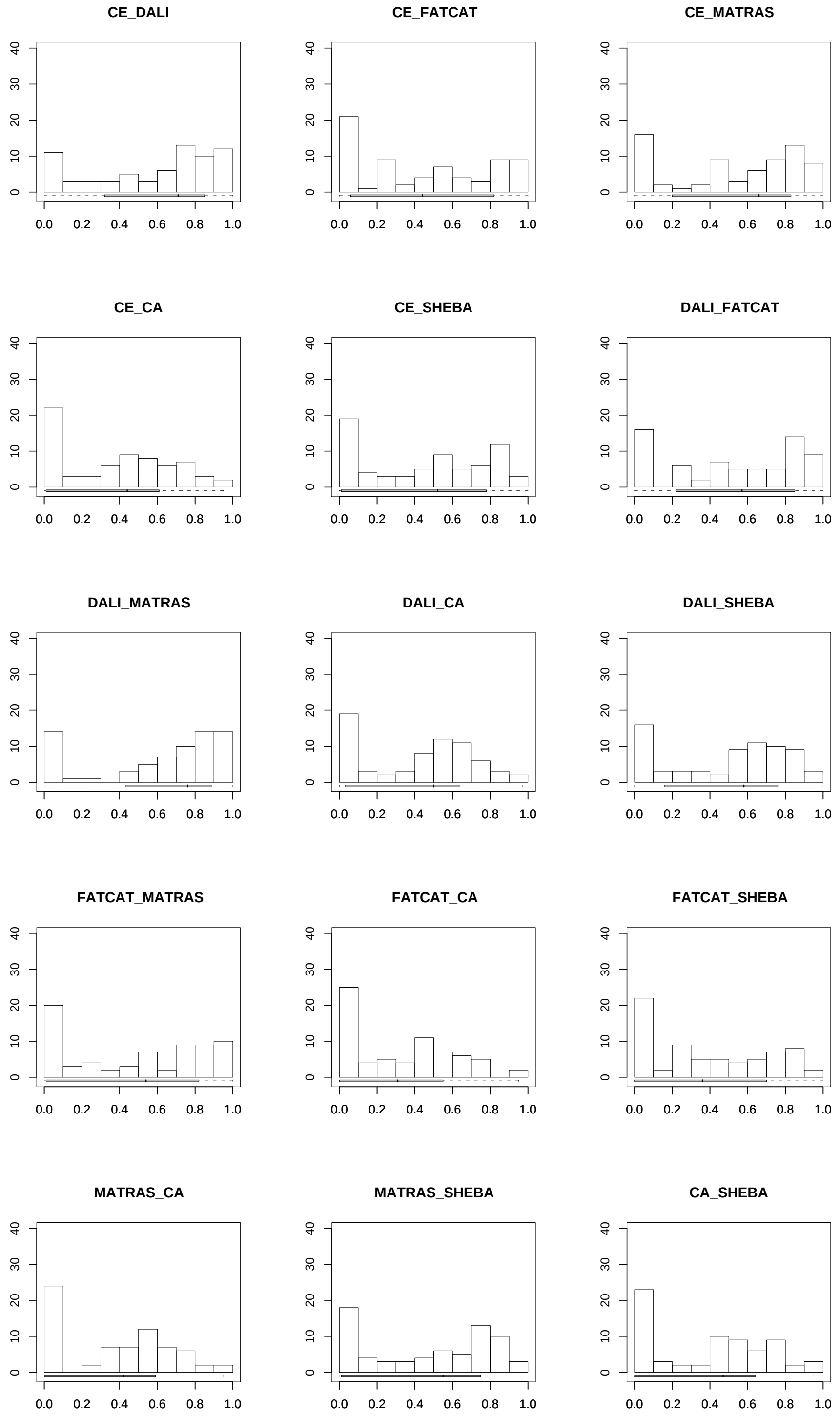

Figure S3: Histograms for the alignment consistency measured by A_0 in SISY set.

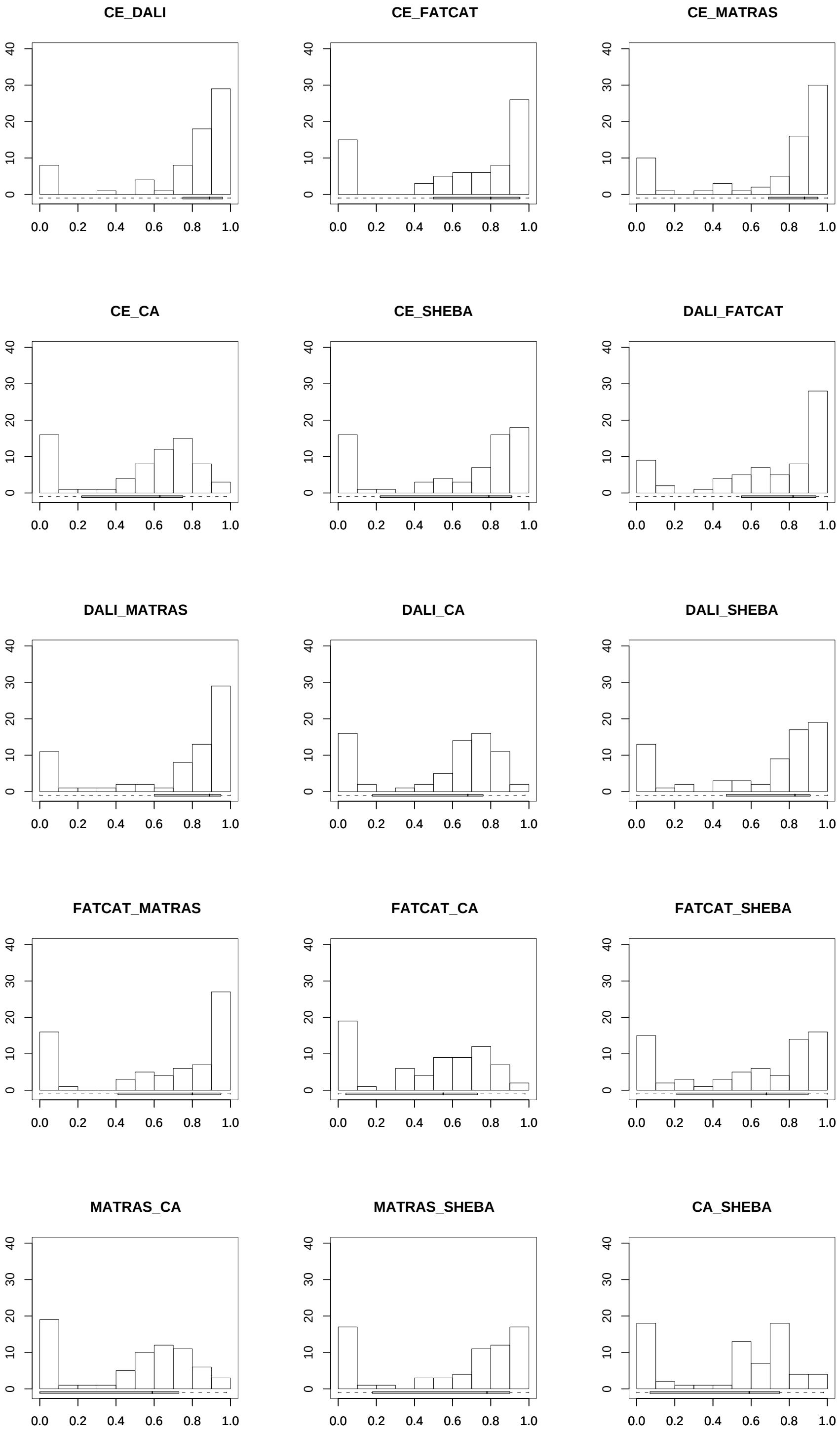

Figure S4: Histograms for the alignment consistency measured by A_4 in SISY set.

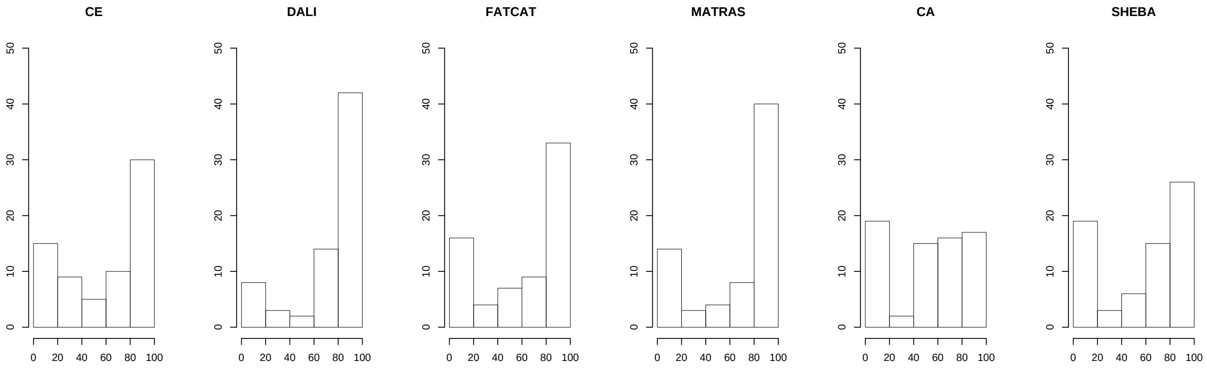

Figure S5: Histograms for the distribution of the percentages of agreement to the reference alignments in the SISY set.
